# The Nonlinear Association Between Adipose Tissue Insulin Resistance and Remnant Cholesterol in Chinese Adults: A Cross-Sectional Study

**DOI:** 10.3390/jcdd13090459

**Published:** 2026-09-11

**Authors:** Ruixiang Cui, Honglin Sun, Mengge Yang, Ying Wang, Jia Liu, Guang Wang

**Affiliations:** 1 Department of Endocrinology, Beijing Chao-Yang Hospital, Capital Medical University, No. 8, Gongti South Road, Chaoyang District, Beijing 100020, China; crx2021@mail.ccmu.edu.cn (R.C.);; 2 Health Management Center, Beijing Chao-Yang Hospital, Capital Medical University, No. 8, Gongti South Road, Chaoyang District, Beijing 100020, China

**Keywords:** adipose tissue, insulin resistance, remnant cholesterol, nonlinear association

## Abstract

**Background**: Adipose tissue insulin resistance (Adipo-IR) is a key driver in the pathogenesis of cardiometabolic diseases. Dyslipidemia, particularly elevated remnant cholesterol (RC), is also strongly associated with cardiometabolic risk and has been recognized as a highly atherogenic lipid marker. Although systemic IR is known to be linked to dyslipidemia and elevated RC, the specific relationship between Adipo-IR and RC remains unclear. This study aimed to investigate the association between Adipo-IR and RC as well as its consistency across different subgroups. **Methods**: A cross-sectional study was conducted involving 6299 Chinese subjects. Logistic regression was used to assess the associations between Adipo-IR quartiles and elevated calculated RC levels. Restricted cubic splines (RCS) and a two-piecewise model were employed to characterize the dose–response relationship. Subgroup analyses were performed by age, sex, and body mass index (BMI). **Results**: Participants in higher Adipo-IR quartiles exhibited a significantly increased risk of elevated RC, low-density lipoprotein cholesterol (LDL-C), and triglycerides (TG) (all *p* for trend <0.001). After full adjustment, the odds ratios (95% CI) for the highest versus lowest Adipo-IR quartile were 1.90 (1.58–2.29) for RC, 1.76 (1.47–2.12) for LDL-C, and 4.74 (3.85–5.86) for TG. RCS analysis revealed a nonlinear increasing relationship between Adipo-IR and RC (*p* for nonlinearity <0.001). An estimated inflection point was identified at an Adipo-IR value of 44.85, with a significant positive association below this point (OR = 1.020, *p* < 0.001) but not above it (OR = 0.997, *p* = 0.248). This positive association was generally consistent across age, sex, and BMI subgroups, and interaction tests were not statistically significant except for the Q3 comparison by sex. **Conclusions**: Adipo-IR showed a nonlinear association with elevated calculated RC, with a positive association below the estimated inflection point of 44.85 in this cohort of Chinese adults.

## 1. Introduction

As an important endocrine organ, adipose tissue plays an important role in maintaining the metabolic balance of the human body [1]. One of the typical manifestations of adipose tissue malfunction is the decrease in the tissue’s sensitivity to insulin, which is also called adipose insulin resistance (Adipo-IR) [2]. Adipo-IR is the pathological mechanism of various cardiometabolic diseases such as type 2 diabetes (T2D), metabolic dysfunction-associated steatotic liver disease (MASLD), coronary heart disease, and hypertension [3,4,5,6].

Triglyceride-rich lipoproteins (TRLs) encompass very-low-density lipoproteins (VLDLs), intermediate-density lipoproteins (IDLs), and chylomicron remnants [7]. The cholesterol found in TRLs and the products of their metabolism are known as remnant cholesterol (RC) [8]. RC initially emerged as a major causal risk factor for atherosclerotic cardiovascular disease [9]. Later, elevated RC was gradually found to be linked with the prevalence of obesity, metabolic syndrome, MASLD and T2D [10,11,12,13]. Recently, our team confirmed the strong relationship between elevated RC levels and advanced cardiovascular–kidney–metabolic (CKM) syndrome stages [14]. This further elucidates the complex pathophysiological relationship between RC and cardiometabolic diseases.

While the association between IR assessed by conventional markers such as HOMA-IR and elevated RC levels has been established by some studies, the specific relationship between Adipo-IR and RC remains insufficiently explored. Unlike HOMA-IR which primarily reflects IR based on fasting glucose and insulin levels, Adipo-IR provides information on adipose tissue IR by reflecting impaired insulin-mediated suppression of adipose tissue lipolysis. Therefore, Adipo-IR may provide complementary information beyond conventional IR markers and may offer additional insight into the contribution of adipose tissue dysfunction to RC metabolism. Our study aimed to investigate the association between Adipo-IR and RC as well as its consistency across different subgroups.

## 2. Materials and Methods

### 2.1. Study Population

Participants in this study were at least eighteen years old and must have had a routine physical examination performed at Beijing Chao-Yang Hospital’s health medical center in 2023. Exclusion criteria were defined as follows: (1) currently taking lipid-lowering agents; (2) presence of severe renal or hepatic dysfunction (eGFR < 60 mL/min/1.73 m^2^; ALT or AST > 3 times the upper limit of normal); (3) known history of cardiovascular disease (including coronary artery disease, heart failure, or stroke); (4) pregnancy or lactation; (5) missing data for lipid profiles. Ultimately, 6299 participants were included in our study. The Beijing Chao-Yang Hospital Ethics Committee approved the study procedure, and all subjects submitted informed consent in written form.

### 2.2. Measurement of Clinical Information

Standard procedures were used to measure various blood biochemical parameters at the clinical laboratories of Beijing Chao-Yang Hospital. These parameters included aspartate transaminase (AST), alanine transaminase (ALT), creatinine, fasting blood glucose (FBG), fasting insulin (FINS), hemoglobin A1c (HbA1c), total cholesterol (TC), triglycerides (TG), low-density lipoprotein cholesterol (LDL-C), and high-density lipoprotein cholesterol (HDL-C). We used the glucose oxidase method to measure FBG, chemiluminescence method for FINS, and colorimetric enzymatic method for ALT, AST, creatinine, LDL-C, HDL-C, TC, TG, and free fatty acids (FFAs) (Siemens Healthcare Diagnostics). RC (mmol/L) was calculated as TC (mmol/L) − LDL-C (mmol/L) − HDL-C (mmol/L) [15]. eGFR was calculated by the Chronic Kidney Disease Epidemiology Collaboration (CKD–EPI) equation. The Adipo-IR index was calculated as the product of FINS and FFA concentration (Adipo-IR index = FINS (μIU/mL) × FFA (mmol/L)) [16]. Homeostasis model assessment of insulin resistance (HOMA-IR) was calculated using the formula described by Matthews (HOMA-IR = FINS (µIU/mL) × FBG (mmol/L)/22.5) [17]. Hypertension was diagnosed if systolic blood pressure (SBP) was ≥140 mmHg or diastolic blood pressure (DBP) was ≥90 mmHg measured on two different days [18]. RC was categorized into quartiles according to its distribution in the study population (Q1: RC ≤ 0.39 mmol/L, Q2: 0.39 < RC ≤ 0.58 mmol/L, Q3: 0.58 < RC ≤ 0.81 mmol/L, and Q4: RC > 0.81 mmol/L). There is no universally established clinical cutoff for high RC, so elevated RC was defined as values in the top quartile (Q4) of its distribution within the study population. For TG and LDL-C, elevated levels were also defined as values in the Q4 quartile.

### 2.3. Statistical Analysis

In the present study, the Shapiro–Wilk test was used to test the normality of the variables. Continuous variables with normal distribution were expressed as mean ± standard deviation. Categorical variables were summarized as frequencies and percentages. Differences across RC quartiles were assessed using one-way ANOVA for normally distributed continuous variables, and the chi-square test for categorical variables. Spearman correlation analysis was used to analyze the coefficient of correlation between Adipo-IR and RC. The association between Adipo-IR quartiles and dyslipidemia was evaluated using multivariable logistic regression analysis, with the lowest quartile (Q1) serving as the reference group. The results are presented as odds ratios (ORs) and 95% confidence intervals (CIs). The dose–response relationship between Adipo-IR and RC levels was examined using restricted cubic spline (RCS) regression with three knots placed at the 10th, 50th, and 90th percentiles of Adipo-IR. Three knots were selected to provide sufficient flexibility for modeling potential nonlinear associations while minimizing the risk of overfitting. Threshold effects were assessed using a two-piecewise logistic regression model to identify the potential inflection point in the association between Adipo-IR and elevated RC, with a ΔAIC > 2 indicating a better model fit, and bootstrap resampling was performed to estimate the uncertainty of the inflection point. For trend analyses, the median value of Adipo-IR within each quartile was assigned to participants in the corresponding quartile and entered into the regression model as a continuous variable, and the resulting *p* value was reported as the *p* for trend. Subgroup analyses stratified by age, sex, and BMI were performed, and multiplicative interaction terms were included to test for effect modification. The *p* for interaction was calculated by including multiplicative interaction terms in the regression models. All statistical analyses were conducted using R version 4.5.1.

## 3. Results

### 3.1. Baseline Characteristics by RC Quartiles

As shown in Table 1, 6299 subjects were included in the study. As the calculated RC quartile increased, the proportion of males decreased from 52.9% to 37%, while the average age of participants ranged from 42.92 to 48.74 years and the average BMI increased from 23.86 to 25.72 kg/m^2^. Participants in higher RC quartiles tended to be older and have higher body weight, BMI, systolic and diastolic blood pressure, and worse lipid profiles, including elevated TC, TG, and reduced HDL-C. There was no apparent increasing or decreasing trend in LDL-C levels across Q1–Q4. Liver enzymes (AST and ALT), glycemic markers (HbA1c and FBG) and insulin resistance indices (Adipo-IR and HOMA-IR) also showed an increasing trend across RC quartiles. In contrast, eGFR decreased with higher RC levels. Significant differences were observed in all continuous and categorical variables among the four RC quartiles.

### 3.2. Association Between Adipo-IR Quartiles and Dyslipidemia

Given that various types of blood lipids increased as the quantile levels of Adipo-IR increased, we used logistic regression analyses to assess the relationship between Adipo-IR and increases in the three lipid components separately (Table 2). In the crude models, the ORs of the highest Adipo-IR quartile for elevated RC, LDL-C and TG were 2.61 (95%CI 2.21–3.09), 2.28 (95%CI 1.94–2.70) and 7.11 (95%CI 5.89–8.63) respectively (all *p* for trend <0.001). After adjusting for age, sex, BMI, HbA1c, eGFR, and hypertension, the ORs of the highest Adipo-IR quartile for elevated RC, LDL-C and TG were 1.90 (1.58–2.29), 1.76 (1.47–2.12) and 4.74 (3.85–5.86) respectively (all *p* for trend <0.001). We found that the risk of elevation of these three lipid components all increased across Adipo-IR quartiles. This suggested that Adipo-IR was closely associated with lipid metabolism dysregulation.

### 3.3. Nonlinear Association Between Adipo-IR and Elevated RC

**Figure 1:**
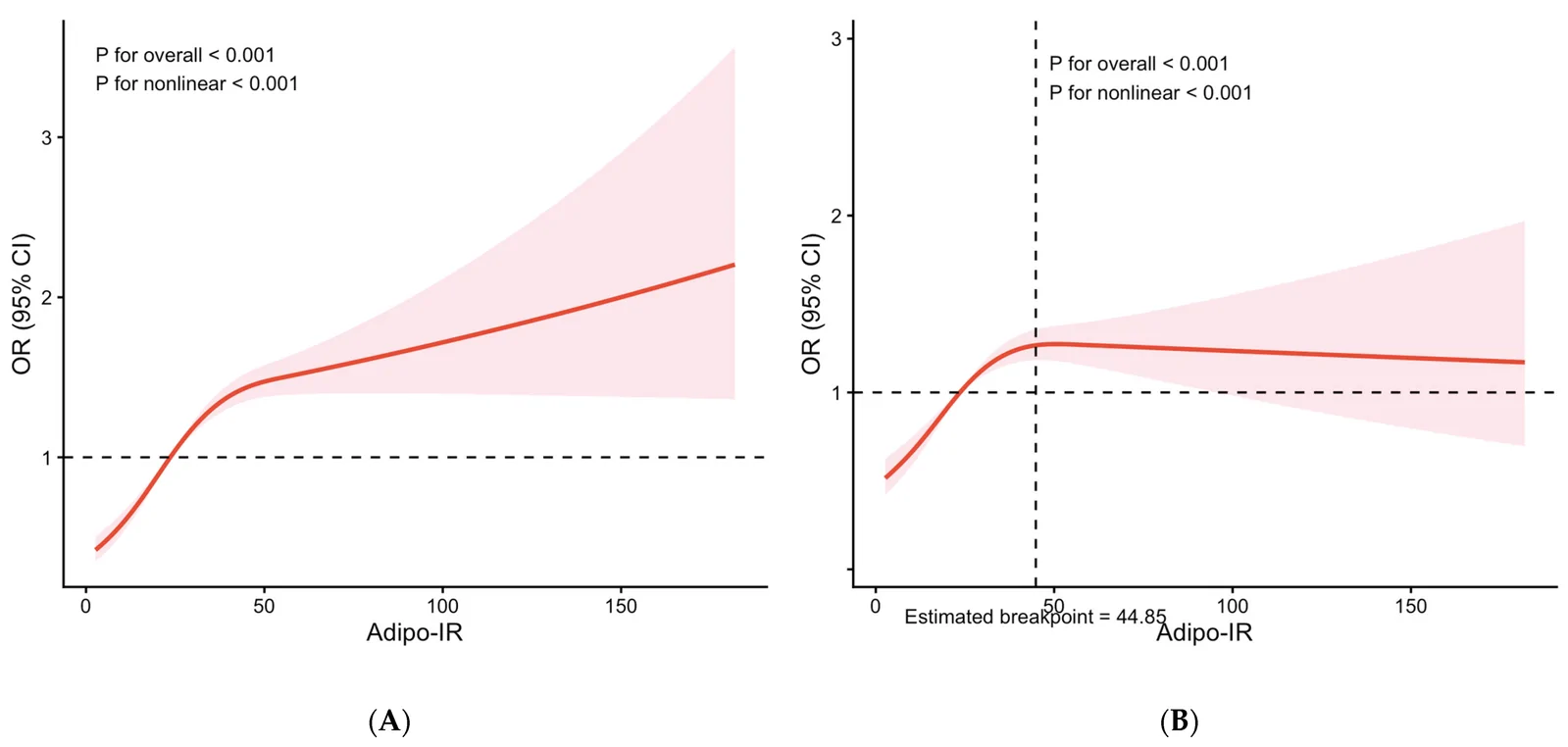
Nonlinear association between Adipo-IR and elevated RC risk. (**A**) Unadjusted RCS curve. (**B**) RCS curve adjusted for age, sex, BMI, HbA1c, eGFR, and hypertension. The solid line represents the OR, and the dashed lines represent the 95% CI. The reference line (OR = 1) is set at the median Adipo-IR value. *p* values for the overall association and nonlinearity are shown.

Next, we focused on figuring out whether this association between Adipo-IR and RC was linear or followed a more complex pattern. Restricted cubic spline (RCS) analysis revealed a significant nonlinear dose–response relationship between Adipo-IR and the risk of elevated RC (*p* for nonlinearity <0.001) (Figure 1). In the crude model (Figure 1A), a significant nonlinear relationship between Adipo-IR and the odds of high RC was found (*p* for overall <0.001, *p* for nonlinearity <0.001). After further adjustment for age, sex, BMI, HbA1c, eGFR, and hypertension (Figure 1B), the nonlinear association remained robust and statistically significant (*p* for overall <0.001, *p* for nonlinearity <0.001).

A threshold effect was identified at an Adipo-IR value of 44.85 using a two-piecewise logistic regression model (Table 3). The bootstrap analysis yielded a 95% confidence interval of 19.08–67.34. Below this inflection point, each unit increase in Adipo-IR was significantly associated with a 2.0% increase in the odds of elevated RC (OR = 1.020, 95% CI: 1.013–1.027, *p* < 0.001). Above this threshold, however, the association was no longer statistically significant (OR = 0.997, 95% CI: 0.992–1.002, *p* = 0.248). The two-piecewise model provided a better fit than the standard linear model (ΔAIC = 28.19), further supporting a nonlinear association.

### 3.4. Associations Between Adipo-IR and RC in Subgroups Stratified by Age, Sex, and BMI

**Figure 2:**
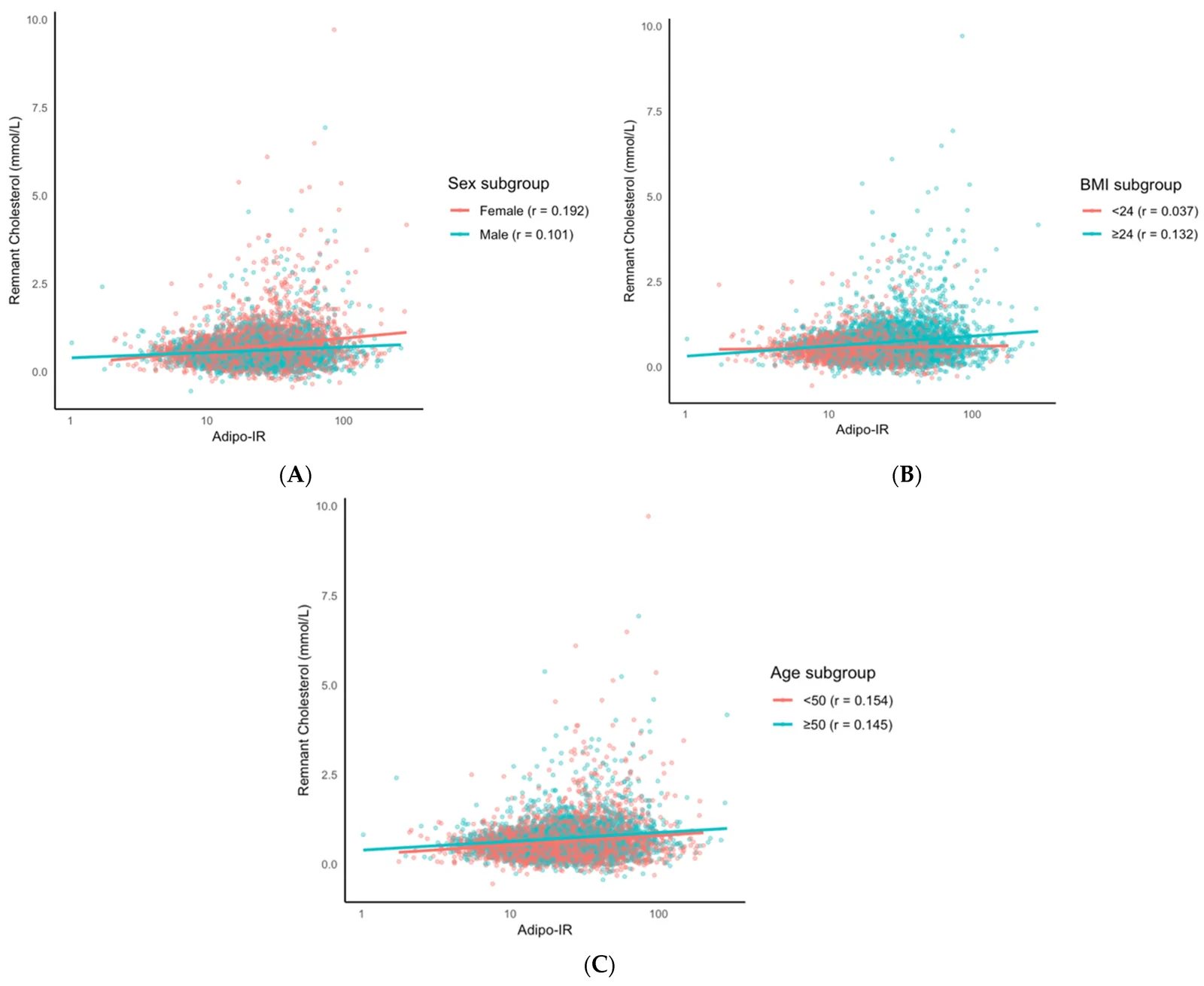
Correlation between Adipo-IR and RC across demographic subgroups. Scatter plots display Spearman correlation analyses stratified by (**A**) sex, (**B**) BMI, and (**C**) age. The Spearman correlation coefficient (r) for each subgroup is indicated on the plot.

To examine whether the link between Adipo-IR and RC was consistent across different populations, we performed stratified correlation analyses (Figure 2). Spearman correlation analyses revealed positive associations between Adipo-IR and RC in all stratifications. Correlations were observed in both females (r = 0.192) and males (r = 0.101), with a potentially stronger association in females (Figure 2A). Consistent correlations were evident in both the BMI < 24 kg/m^2^ group (r = 0.037) and those who are overweight or obese (r = 0.132) (Figure 2B). The positive association was maintained in both younger (<50 years) and older (≥50 years) adults (r = 0.154 and 0.145 respectively) (Figure 2C). These stratified analyses demonstrate that the positive relationship between Adipo-IR and RC is robust across sex, age, and BMI subgroups.

To further clarify the relationship between Adipo-IR and RC in different subgroups, we adjusted covariates and performed logistic regression analysis in each group (Table 4). After adjusting for age, BMI, HbA1c, eGFR, and hypertension, ORs of elevation RC were significantly higher in the Q4 quartiles than in the Q1 quartiles in both men and women (*p* < 0.001 and *p* = 0.004 respectively). A statistically significant interaction by sex was observed specifically for Q3 (P for interaction = 0.007), with a stronger association observed in women (OR = 1.88, 95% CI: 1.49–2.38) than in men (OR = 1.11, 95% CI: 0.83–1.50). However, the interaction was not statistically significant for Q2 or Q4, indicating no significant sex difference in the associations.

After adjusting for sex, BMI, HbA1c, eGFR, and hypertension, those in the highest Adipo-IR quartile had 1.91-fold increased odds (OR = 1.91, 95% CI: 1.48–2.47) of high RC compared to those in the lowest Adipo-IR quartile in participants younger than 50 years old. The ORs were also significantly higher in the Q3 quartile (OR = 1.58, 95% CI: 1.24–2.03). In those aged 50 and above, a positive association was also present, with the Q4 quartile showing 1.91-fold higher odds (OR = 1.91, 95% CI: 1.45–2.52) than Q1.

After adjusting for sex, age, HbA1c, eGFR, and hypertension, we found that in individuals with BMI < 24 kg/m^2^, the odds of high RC in the Q2 (OR = 1.34, 95% CI: 1.03–1.74), Q3 (OR = 1.50, 95% CI: 1.13–2.00) and Q4 (OR = 1.60, 95% CI: 1.14–2.22) quartiles were 1.34, 1.50 and 1.60 times greater than those in the Q1 Adipo-IR quartile. Among individuals with BMI ≥ 24 kg/m^2^, a positive association was also observed, with the Q4 quartile conferring a 2.11-fold increase in odds (OR = 2.11, 95% CI: 1.66–2.68) compared to Q1. No significant BMI–Adipo-IR interaction was detected. 

**Figure 3:**
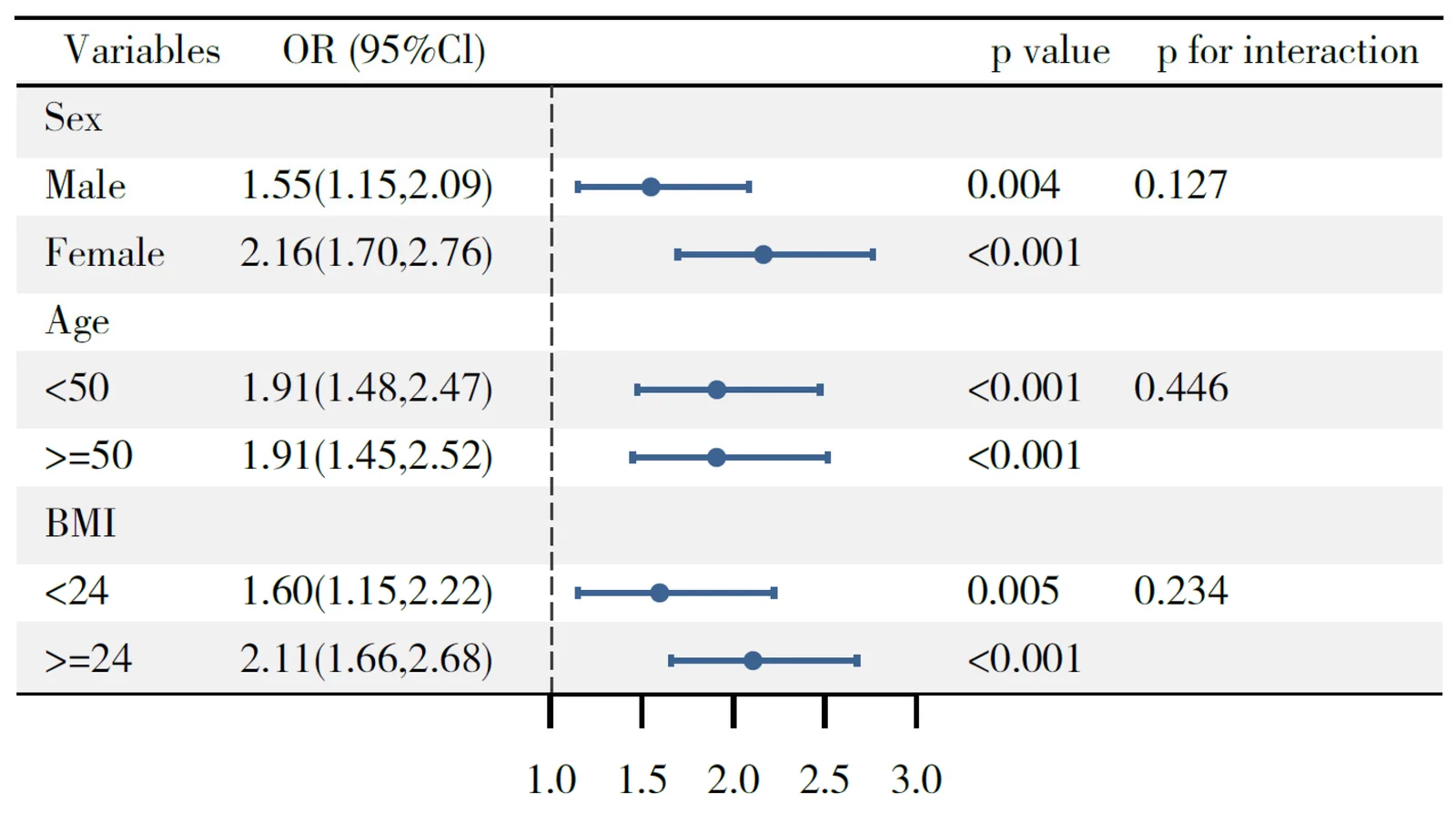
Forest plot of the association between high Adipo-IR and elevated RC in subgroups. Odds ratios (ORs) and 95% confidence intervals for the highest versus lowest Adipo-IR quartile are shown. Analyses are stratified by age, sex, and BMI categories. The first quartile (Q1) was the reference group. When sex was stratified, age, BMI, HbA1c, eGFR, and hypertension were adjusted; when age was stratified, sex, BMI, HbA1c, eGFR, and hypertension were adjusted; when BMI was stratified, age, sex, HbA1c, eGFR, and hypertension were adjusted. *p* for interaction values for sex, age, and BMI subgroups is shown next to each corresponding subgroup.

To visually present the potential effect modification of the association between Adipo-IR and elevated RC across key demographic subgroups, we constructed a forest plot based on the fully adjusted logistic regression models (Figure 3). The forest plot graphically confirmed the robust positive association between the highest Adipo-IR quartile (Q4) and the risk of elevated RC in nearly all subgroups.

The first quartile (Q1) was the reference group. When sex was stratified, age, BMI, HbA1c, eGFR, and hypertension were adjusted; when age was stratified, sex, BMI, HbA1c, eGFR, and hypertension were adjusted; and when BMI was stratified, age, sex, HbA1c, eGFR, and hypertension were adjusted.

## 4. Discussion

Unlike previous research that mainly emphasized the effects of Adipo-IR or RC on adverse cardiovascular events, our cross-sectional study focused on the direct association between Adipo-IR and calculated RC for the first time. We found that Adipo-IR was independently associated with an atherogenic lipid profile, particularly with elevated RC, in a nonlinear manner. 

The lipid profile was found largely influenced by IR. Kron et al. discovered alterations in lipid metabolism, including HDL-C and LDL-C, when HOMA-IR surpassed this cutoff value of 3.63 [19]. Both TyG, the surrogate index of IR, and HOMA-IR have been reported as reliable indicators of dyslipidemia [20,21]. Specifically, Adipo-IR was found positively correlated with the levels of TG, LDL-C and VLDL, and negatively correlated with the level of HDL-C in a US-based study [22]. Beyond these traditional lipid parameters, dyslipidemia mediates its atherogenic effects also through the accumulation of RC, which has become increasingly concerned. Some previous studies have explored the relationship between IR and RC. In a non-diabetic Japanese population, RC levels were found significantly higher in insulin-resistant individuals than in insulin-sensitive individuals and were positively correlated with HOMA-IR independent of age, sex, and BMI [23]. In both a non-obese diabetic population and patients with MASLD, significant positive correlations between HOMA-IR and RC were found [24,25]. Li et al. found that RC was positively correlated with HOMA-IR, and IR mediated the link between RC and T2D [26].

Unlike previous studies focusing on RC and IR assessed by HOMA-IR, our study specifically focused on Adipo-IR. Whereas HOMA-IR which primarily reflects IR based on fasting glucose and insulin levels, Adipo-IR provides information on adipose tissue IR by reflecting impaired insulin-mediated suppression of lipolysis and the consequent increase in FFA flux to the liver. Given the role of hepatic FFA flux in VLDL production, Adipo-IR may provide complementary information on RC metabolism that is not fully captured by HOMA-IR. As the primary driver of systemic IR, Adipo-IR impairs adipose tissue–organ crosstalk, thereby contributing to a spectrum of cardiometabolic diseases, including coronary heart disease, heart failure, hepatic steatosis, and islet dysfunction [27]. However, the link between IR specifically in adipose tissue and RC has not been established in the Chinese population yet. Thus, investigating Adipo-IR may have potential utility for identifying individuals with a prominent adipose tissue insulin-resistant phenotype, potentially complementing conventional measures for metabolic risk stratification. Prospective studies are needed to determine whether it provides incremental clinical value beyond HOMA-IR and whether incorporating Adipo-IR into routine risk assessment can improve patient management. Of note, our Q4 cutoff of 0.81 mmol/L for elevated RC may vary across populations, as it was not based on a universally established clinical threshold. Nevertheless, this cutoff was broadly comparable to upper-quartile cutoffs reported in previous studies, suggesting reasonable comparability with prior epidemiological evidence [28,29].

Insulin maintains lipid homeostasis by inhibiting the synthesis and secretion of very-low-density lipoprotein (VLDL) in the liver. Michel et al. found that cholesterol synthesis metabolism was greater but cholesterol absorption was weaker in insulin-resistant patients than in insulin-sensitive ones, thus inferring a trend towards increased synthesis but decreased absorption of hepatic cholesterol under the condition of IR [30]. In the presence of IR, excessive VLDL production may contribute to elevated RC levels. In addition, reduced intestinal cholesterol absorption may trigger compensatory hepatic secretory mechanisms that further increase VLDL production. Insulin suppresses lipolysis via decreasing cAMP in a way that is dependent on AKT/PDE3B [2]. When insulin sensitivity in adipose tissue declines, insulin inhibition of lipolysis in adipose tissue is weakened. Free fatty acids, the byproduct of lipolysis, are thus overproduced, which directly stimulated the synthesis of VLDL in the liver [31]. Impairment in the clearance of TRL remnants also contributes to the accumulation of RC. As lipoprotein lipase (LPL) is the master regulator of TRL lipolysis [32], its suppression in the state of IR—as demonstrated by Panarotto et al.—may account for this reduced clearance [33]. Therefore, it is likely that Adipo-IR contributes to the increase in blood RC levels through these modes of metabolic switching.

The observed nonlinear saturation association between Adipo-IR and RC may have several biological explanations. One possible mechanism involves alterations in insulin signaling and hepatic lipid metabolism. Insulin signaling through Akt can suppress AMPK activity [34]. AMPK has been shown to directly phosphorylate and inhibit the transcriptional activity of sterol regulatory element-binding protein-1c (SREBP-1c), thereby curbing hepatic lipid synthesis [35]. In the early stage of Adipo-IR, compensatory hyperinsulinemia may promote hepatic lipogenesis through enhanced insulin signaling, potentially promoting VLDL production and contributing to higher RC levels. However, with progressive metabolic deterioration, increasing IR may alter the relationship between insulin signaling and hepatic lipid metabolism, such that further increases in Adipo-IR may not translate into increases in hepatic lipogenesis and VLDL production. This may contribute to the lack of further increase in RC at higher levels of Adipo-IR. Importantly, the estimated inflection point of 44.85 should not be interpreted as a clinically established threshold. Rather, it represents a data-derived statistical inflection point within the present study population. Although it may indicate a change in the strength of the association between Adipo-IR and elevated RC, its biological and clinical relevance remains uncertain and requires validation in independent populations.

Subgroup analysis showed that the association between adipose tissue IR and elevated RC may be relatively consistent across different age, BMI and sex groups. Across age strata, the ORs for elevated RC were broadly comparable at each Adipo-IR quartile, with similar effect estimates observed between participants aged <50 and ≥50 years. This consistency across individual Adipo-IR quartiles may reflect the fundamental role of adipose tissue insulin resistance in regulating FFA flux and hepatic lipid metabolism across different stages of adulthood. In contrast, the associations appeared somewhat stronger in women than in men and in participants with BMI ≥ 24 kg/m^2^ than in those with BMI < 24 kg/m^2^. However, formal interaction testing did not provide consistent evidence of effect modification by sex or BMI, except for a statistically significant interaction at the Q3 level for sex. These observed differences may have a biological basis, as obesity is characterized by adipose tissue dysfunction, including enhanced basal lipolysis, inflammation, and reduced adiponectin secretion [36,37], while previous studies have reported sex-related differences in adipose tissue insulin sensitivity and lipid storage capacity [38]. Such alterations may affect FFA flux and hepatic lipid metabolism and could potentially contribute to heterogeneity in the Adipo-IR–RC association across individuals. However, given the lack of consistent statistical evidence for interaction, these potential biological explanations should be considered hypothesis-generating and interpreted cautiously.

There are several limitations to our study. Firstly, due to the cross-sectional nature of our study design, we cannot establish a causal relationship between Adipo-IR and elevated RC. Secondly, although we adjusted for multiple potential confounders, residual confounding from unmeasured factors (e.g., dietary habits, physical activity levels, smoking, alcohol consumption, lipid-lowering and glucose-lowering medication use, lifestyle interventions, and genetic predispositions) cannot be entirely ruled out. Thirdly, our single-center design and recruitment from a health examination center may introduce selection bias, limiting representativeness. Fourthly, RC was calculated by formula rather than directly measured, which may affect the precision of RC estimation and limit direct comparability with studies using direct measurement methods. Fifthly, the highest quartile cutoff for RC was population-specific and may not be directly comparable across different studies. Lastly, our findings from a Chinese population may not be generalizable to other ethnic groups.

## 5. Conclusions

To sum up, we found that Adipo-IR showed a nonlinear association with elevated calculated RC, with a positive association below the estimated inflection point of 44.85 in this cohort of Chinese adults. The finding underscored Adipo-IR as a factor significantly associated with RC-related cardiometabolic risk.

## Figures and Tables

**Table 1:** Baseline characteristics by RC quartiles.

Variable	Q1 RC ≤ 0.39 mmol/L	Q2 0.39 < RC ≤ 0.58 mmol/L	Q3 0.58 < RC ≤ 0.81 mmol/L	Q4 RC > 0.81 mmol/L	*p* Value
	(n = 1575)	(n = 1575)	(n = 1575)	(n = 1574)	
Sex, male, n (%)	833 (52.9%)	808 (51.3%)	759 (48.2%)	583 (37%)	<0.001
Age, years	42.92 ± 13.67	42.98 ± 13.55	44.93 ± 13.22	48.74 ± 12.01	<0.001
Weight, kg	66.88 ± 13.53	66.86 ± 13.08	68.51 ± 13.78	72.64 ± 12.95	<0.001
Height, cm	166.97 ± 8.36	166.77 ± 8.37	166.87 ± 8.85	167.67 ± 8.82	0.015
BMI, kg/m^2^	23.86 ± 3.75	23.93 ± 3.68	24.47 ± 3.79	25.72 ± 3.38	<0.001
Systolic BP, mmHg	121.15 ± 18.24	120.25 ± 17.09	122.7 ± 17.83	127.81 ± 18.16	<0.001
Diastolic BP, mmHg	71.74 ± 11.84	71.06 ± 11.56	72.54 ± 11.46	76.08 ± 12.08	<0.001
Hypertension, %	266 (16.9%)	229 (14.5%)	280 (17.8%)	401 (25.5%)	<0.001
TC, mmol/L	4.81 ± 0.92	4.75 ± 0.86	5.00 ± 0.87	5.50 ± 1.02	<0.001
HDL-C, mmol/L	1.40 ± 0.35	1.40 ± 0.34	1.35 ± 0.35	1.19 ± 0.31	<0.001
LDL-C, mmol/L	3.18 ± 0.92	2.87 ± 0.82	2.97 ± 0.8	3.04 ± 0.91	<0.001
TG, mmol/L	1.03 ± 0.42	1.15 ± 0.48	1.39 ± 0.56	2.83 ± 2.45	<0.001
AST, U/L	22.48 ± 12.1	22.77 ± 8.69	23.43 ± 12.83	25.19 ± 25.6	<0.001
ALT, U/L	22.74 ± 16.71	23.14 ± 17.07	24.6 ± 21.08	28.71 ± 24.67	<0.001
HbA1c, %	5.59 ± 0.7	5.54 ± 0.72	5.61 ± 0.73	5.83 ± 1	<0.001
FBG, mmol/L	4.96 ± 1.14	5 ± 1.19	5.12 ± 1.25	5.51 ± 1.73	<0.001
FINS, uIU/mL	9 ± 5.65	8.7 ± 6.1	8.86 ± 5.45	10.4 ± 6.15	<0.001
eGFR, mL/min per 1.73 m^2^	107.5 ± 20.33	104.55 ± 24.34	102.84 ± 22.84	97.73 ± 24.21	<0.001
Adipo-IR	28.84 ± 21.75	26.9 ± 21.22	27.77 ± 22.12	34.51 ± 24.74	<0.001
HOMA-IR	2.05 ± 1.84	1.99 ± 1.7	2.08 ± 1.64	2.59 ± 1.89	<0.001

Adipo-IR, adipose tissue insulin resistance; AST, aspartate transaminase; ALT, alanine transaminase; BMI, body mass index; BP, blood pressure; eGFR, estimated glomerular filtration rate; FBG, fasting blood glucose; FINS, fasting insulin; HbA1c, hemoglobin A1c; HDL-C, high-density lipoprotein cholesterol; HOMA-IR, homeostasis model assessment of insulin resistance; LDL-C, low-density lipoprotein cholesterol; TG, triglycerides; TC, total cholesterol.

**Table 2:** Logistic regression between Adipo-IR and blood lipid profile.

Variables	RC		LDL-C		TG	
	Model 1 OR (95%CI)	Model 2 OR (95%CI)	Model 1 OR (95%CI)	Model 2 OR (95%CI)	Model 1 OR (95%CI)	Model 2 OR (95%CI)
Q1	Ref.	Ref.	Ref.	Ref.	Ref.	Ref.
Q2	1.41 (1.18–1.69) ***	1.33 (1.11, 1.60) **	1.46 (1.23–1.74) ***	1.39 (1.17, 1.66) ***	1.97 (1.60–2.42) ***	1.86 (1.50, 2.31) ***
Q3	1.74 (1.46–2.07) ***	1.52 (1.27, 1.83) ***	1.57 (1.33–1.87) ***	1.41 (1.18, 1.69) ***	3.14 (2.58–3.83) ***	2.64 (2.15, 3.26) ***
Q4	2.61 (2.21–3.09) ***	1.90 (1.58, 2.29) ***	2.28 (1.94–2.70) ***	1.76 (1.47, 2.12) ***	7.11 (5.89–8.63) ***	4.74 (3.85, 5.86) ***
*p* for trend	<0.001	<0.001	<0.001	<0.001	<0.001	<0.001

Logistic regression analysis showing the association between Adipo-IR quartiles and elevated lipid levels. Elevated levels of RC, LDL-C, and TG were defined as values in the top quartile (Q4) of their distribution. Model 1 was unadjusted. Model 2 was adjusted for age, sex, BMI, HbA1c, eGFR, and hypertension. Q1 was used as the reference group. OR, odds ratio; CI, confidence interval. ** *p* < 0.01; *** *p* < 0.001.

**Table 3:** Two-piecewise logistic regression analysis of the association between Adipo-IR and elevated RC.

	Adjusted OR (95% CI)	*p*-Value
Fitting by the standard logistic model	1.006 (1.004, 1.009)	<0.001
Fitting by the two-piecewise logistic model		
Inflection point	44.85 (19.08, 67.34)	
Adipo-IR < 44.85	1.020 (1.013, 1.027)	<0.001
Adipo-IR > 44.85	0.997 (0.992, 1.002)	0.248
ΔAIC	28.19	

The model was adjusted for age, sex, BMI, HbA1c, eGFR, and hypertension. ΔAIC, the difference in AIC between the standard linear and two-piecewise logistic regression models. OR, odds ratio; CI, confidence interval.

**Table 4:** Logistic regression between Adipo-IR and RC stratified by sex, age and BMI.

	OR (95%CI)	*p* Value	OR (95%CI)	*p* Value	*p* for Interaction
	Female		Male		
Q1	Ref.		Ref.		
Q2	1.49 (1.18, 1.89)	<0.001	1.12 (0.84, 1.51)	0.438	0.170
Q3	1.88 (1.49, 2.38)	<0.001	1.11 (0.83, 1.50)	0.471	0.007
Q4	2.16 (1.70, 2.77)	<0.001	1.55 (1.15, 2.09)	0.004	0.127
	Age < 50 years		Age ≥ 50 years		
Q1	Ref.		Ref.		
Q2	1.23 (0.95, 1.58)	0.112	1.51 (1.15, 1.98)	0.003	0.265
Q3	1.58 (1.24, 2.03)	<0.001	1.48 (1.12, 1.95)	0.006	0.523
Q4	1.91 (1.48, 2.47)	<0.001	1.91 (1.45, 2.52)	<0.001	0.446
	BMI < 24 kg/m^2^		BMI ≥ 24 kg/m^2^		
Q1	Ref.		Ref.		
Q2	1.34 (1.03, 1.74)	0.029	1.33 (1.03, 1.73)	0.032	0.953
Q3	1.50 (1.13, 2.00)	0.005	1.57 (1.22, 2.01)	<0.001	0.844
Q4	1.60 (1.14, 2.22)	0.005	2.11 (1.66, 2.68)	<0.001	0.234

## Data Availability

The datasets generated and/or analyzed during the current study are available from the corresponding author on reasonable request.

## Abbreviations

The following abbreviations are used in this manuscript:

Adipo-IR	Adipose tissue insulin resistance
AIC	Akaike information criterion
ALT	Alanine transaminase
AST	Aspartate transaminase
BMI	Body mass index
BP	Blood pressure
CI	Confidence interval
CKD-EPI	Chronic Kidney Disease Epidemiology Collaboration
CKM	Cardiovascular–kidney–metabolic
DBP	Diastolic blood pressure
eGFR	Estimated glomerular filtration rate
FBG	Fasting blood glucose
FFA	Free fatty acid
FINS	Fasting insulin
HbA1c	Hemoglobin A1c
HDL-C	High-density lipoprotein cholesterol
HOMA-IR	Homeostasis model assessment of insulin resistance
IDL	Intermediate-density lipoproteins
LDL-C	Low-density lipoprotein cholesterol
MASLD	Metabolic dysfunction-associated steatotic liver disease
OR	Odds ratio
Q	Quartile
RC	Remnant cholesterol
RCS	Restricted cubic splines
SBP	Systolic blood pressure
TC	Total cholesterol
TG	Triglycerides
T2D	Type 2 diabetes
TRLs	Triglyceride-rich lipoproteins
VLDLs	Very-low-density lipoproteins

## References

[1] Kershaw E.E., Flier J.S. (2004). Adipose tissue as an endocrine organ. J. Clin. Endocrinol. Metab..

[2] Sancar G., Birkenfeld A.L. (2024). The role of adipose tissue dysfunction in hepatic insulin resistance and T2D. J. Endocrinol..

[3] Savage D.B., Petersen K.F., Shulman G.I. (2007). Disordered lipid metabolism and the pathogenesis of insulin resistance. Physiol. Rev..

[4] Tilg H., Moschen A.R. (2010). Evolution of inflammation in nonalcoholic fatty liver disease: The multiple parallel hits hypothesis. Hepatology.

[5] Rask-Madsen C., Kahn C.R. (2012). Tissue-specific insulin signaling, metabolic syndrome, and cardiovascular disease. Arterioscler. Thromb. Vasc. Biol..

[6] Hall J.E., do Carmo J.M., da Silva A.A., Wang Z., Hall M.E. (2015). Obesity-induced hypertension: Interaction of neurohumoral and renal mechanisms. Circ. Res..

[7] Nordestgaard B.G. (2016). Triglyceride-rich lipoproteins and atherosclerotic cardiovascular disease: New insights from epidemiology, genetics, and biology. Circ. Res..

[8] Varbo A., Benn M., Nordestgaard B.G. (2014). Remnant cholesterol as a cause of ischemic heart disease: Evidence, definition, measurement, atherogenicity, high risk patients, and present and future treatment. Pharmacol. Ther..

[9] Yang X.H., Zhang B.L., Cheng Y., Fu S.K., Jin H.M. (2023). Association of remnant cholesterol with risk of cardiovascular disease events, stroke, and mortality: A systematic review and meta-analysis. Atherosclerosis.

[10] Yu T., Liu S., Fang L., Du T., Liu Z. (2025). Remnant cholesterol in obesity phenotypes: Results from NHANES. Lipids Health Dis..

[11] Li F., Yuan H., Cai S., Piao W., Nan J., Yang Y., Zhao L., Yu D. (2024). Association between remnant cholesterol and metabolic syndrome among Chinese adults: Chinese Nutrition and Health Surveillance (2015–2017). Nutrients.

[12] Hu X., Liu Q., Guo X., Wang W., Yu B., Liang B., Zhou Y., Dong H., Lin J. (2022). The role of remnant cholesterol beyond low-density lipoprotein cholesterol in diabetes mellitus. Cardiovasc. Diabetol..

[13] Miao Y., Tao H. (2023). Association between remnant lipoprotein cholesterol levels and risk of non-alcoholic fatty liver disease in non-obese populations: A Chinese longitudinal prospective cohort study. BMJ Open.

[14] Ding X., Tian J., Chang X., Liu J., Wang G. (2025). Association between remnant cholesterol and the risk of cardiovascular-kidney-metabolic syndrome progression: Insights from the China Health and Retirement Longitudinal Study. Eur. J. Prev. Cardiol..

[15] Mach F., Baigent C., Catapano A.L., Koskinas K.C., Casula M., Badimon L., Chapman M.J., De Backer G.G., Delgado V., Ference B.A. (2020). 2019 ESC/EAS Guidelines for the management of dyslipidaemias: Lipid modification to reduce cardiovascular risk. Eur. Heart J..

[16] Søndergaard E., Espinosa De Ycaza A.E., Morgan-Bathke M., Jensen M.D. (2017). How to measure adipose tissue insulin sensitivity. J. Clin. Endocrinol. Metab..

[17] Matthews D.R., Hosker J.P., Rudenski A.S., Naylor B.A., Treacher D.F., Turner R.C. (1985). Homeostasis model assessment: Insulin resistance and beta-cell function from fasting plasma glucose and insulin concentrations in man. Diabetologia.

[18] Unger T., Borghi C., Charchar F., Khan N.A., Poulter N.R., Prabhakaran D., Ramirez A., Schlaich M., Stergiou G.S., Tomaszewski M. (2020). 2020 International Society of Hypertension global hypertension practice guidelines. J. Hypertens..

[19] Kron V., Verner M., Smetana P., Horáková D., Šlégr J., Studnička F., Bušovský D., Martiník K. (2021). The changes of cholesterol profile at the different insulin resistance range in the Czech Republic. Medicina.

[20] Jiang W.Z., Fan Z.L., Xu M.L., Qian E.H., Lu K.D. (2025). Association between insulin resistance and multiple chronic diseases: A cross-sectional study from CHARLS. J. Health Popul. Nutr..

[21] Day C. (2007). Metabolic syndrome, or What you will: Definitions and epidemiology. Diabetes Vasc. Dis. Res..

[22] Kim J.Y., Bacha F., Tfayli H., Michaliszyn S.F., Yousuf S., Arslanian S. (2019). Adipose tissue insulin resistance in youth on the spectrum from normal weight to obese and from normal glucose tolerance to impaired glucose tolerance to type 2 diabetes. Diabetes Care.

[23] Ohnishi H., Saitoh S., Takagi S., Ohata J.I., Isobe T., Kikuchi Y., Takeuchi H., Shimamoto K. (2002). Relationship between insulin-resistance and remnant-like particle cholesterol. Atherosclerosis.

[24] Taniguchi A., Fukushima M., Sakai M., Miwa K., Makita T., Nagata I., Nagasaka S., Doi K., Okumura T., Fukuda A. (2000). Remnant-like particle cholesterol, triglycerides, and insulin resistance in nonobese Japanese type 2 diabetic patients. Diabetes Care.

[25] Wang S., Zhang Q., Qin B. (2024). Association between remnant cholesterol and insulin resistance levels in patients with metabolic-associated fatty liver disease. Sci. Rep..

[26] Li Y., Zeng Q., Peng D., Hu P., Luo J., Zheng K., Yin Y., Si R., Xiao J., Li S. (2024). Association of remnant cholesterol with insulin resistance and type 2 diabetes: Mediation analyses from NHANES 1999–2020. Lipids Health Dis..

[27] Gilani A., Stoll L., Homan E.A., Lo J.C. (2024). Adipose signals regulating distal organ health and disease. Diabetes.

[28] Yuan L., Liu J., Huang Z., Zhao Y., Feng Y., Yang X., Hu H., Zhang J., Li T., Li Y. (2023). Elevated remnant cholesterol increase 6-year type 2 diabetes mellitus onset risk. Clin. Chim. Acta.

[29] Li Y., Xu Z., Xiao D., Yun X., Liu C., Xiao Q., Li C., Liang Y., Chen D., Du T. (2026). Exploring the relationship between remnant cholesterol and diabetic kidney disease in Chinese type 2 diabetes patients. Front. Nutr..

[30] Hoenig M.R., Sellke F.W. (2010). Insulin resistance is associated with increased cholesterol synthesis, decreased cholesterol absorption and enhanced lipid response to statin therapy. Atherosclerosis.

[31] Boden G. (1997). Role of fatty acids in the pathogenesis of insulin resistance and NIDDM. Diabetes.

[32] Taskinen M.R. (2003). Diabetic dyslipidaemia: From basic research to clinical practice. Diabetologia.

[33] Panarotto D., Rémillard P., Bouffard L., Maheux P. (2002). Insulin resistance affects the regulation of lipoprotein lipase in the postprandial period and in an adipose tissue-specific manner. Eur. J. Clin. Investig..

[34] Hahn-Windgassen A., Nogueira V., Chen C.C., Skeen J.E., Sonenberg N., Hay N. (2005). Akt activates the mammalian target of rapamycin by regulating cellular ATP level and AMPK activity. J. Biol. Chem..

[35] Horton J.D., Goldstein J.L., Brown M.S. (2002). SREBPs: Activators of the complete program of cholesterol and fatty acid synthesis in the liver. J. Clin. Investig..

[36] Sarvari A.K., Van Hauwaert E.L., Markussen L.K., Gammelmark E., Marcher A.-B., Ebbesen M.F., Nielsen R., Brewer J.R., Madsen J.G.S., Mandrup S. (2021). Plasticity of epididymal adipose tissue in response to diet-induced obesity at single-nucleus resolution. Cell Metab..

[37] Stojsavljević S., Gomerčić Palčić M., Virović Jukić L., Smirčić Duvnjak L., Duvnjak M. (2014). Adipokines and proinflammatory cytokines, the key mediators in the pathogenesis of nonalcoholic fatty liver disease. World J. Gastroenterol..

[38] Karastergiou K., Smith S.R., Greenberg A.S., Fried S.K. (2012). Sex differences in human adipose tissues: The biology of pear shape. Biol. Sex. Differ..

